# Sucralose consumption ameliorates high-fat diet-induced glucose intolerance and liver weight gain in mice

**DOI:** 10.3389/fnut.2022.979624

**Published:** 2022-09-26

**Authors:** Pamela Pino-Seguel, Omara Moya, Juan Carlos Borquez, Francisco Pino-de la Fuente, Francisco Díaz-Castro, Camila Donoso-Barraza, Miguel Llanos, Rodrigo Troncoso, Roberto Bravo-Sagua

**Affiliations:** ^1^Laboratorio de Investigación en Nutrición y Actividad Física (LABINAF), Instituto de Nutrición y Tecnología de los Alimentos (INTA), Universidad de Chile, Santiago, Chile; ^2^Advanced Center for Chronic Disease (ACCDiS), Universidad de Chile, Santiago, Chile; ^3^Laboratory of Obesity and Metabolism in Geriatrics and Adults (OMEGA), Instituto de Nutrición y Tecnología de los Alimentos (INTA), Universidad de Chile, Santiago, Chile; ^4^Interuniversity Center for Healthy Aging (CIES), Consortium of Universities of the State of Chile (CUECH), Santiago, Chile

**Keywords:** obesity, non-alcoholic fatty liver disease, sucralose, mitochondria, glucose intolerance

## Abstract

Sucralose is one of the most widely used artificial sweeteners used by the food industry to reduce the calorie density of their products. Although broadly regarded as innocuous, studies show contrasting results depending on whether the research subjects are lean or overweight. In this study, we studied the effect of sucralose consumption on glucose homeostasis in a model of obesity. Male C57BL/6J mice were fed *ad libitum* with control or a high-fat diet (HFD) and drank either water or sucralose (0.1 mg/mL) for 8 weeks. To characterize the ensuing metabolic changes, we evaluated weight gain, glucose and pyruvate tolerance, and physical performance. Also, we assessed markers of steatosis and mitochondrial mass and function in the liver. Our results show that sucralose reduced weight gain, glucose, and pyruvate intolerance, and prevented the decrease in physical performance of HFD-fed mice. In the liver, sucralose also had a positive effect, preventing the decrease in mitochondrial mass exerted by HFD. Altogether, our results indicate that in the context of an obesogenic diet, sucralose has a beneficial effect at the organismal and hepatic levels.

## Introduction

Obesity is a major risk factor for metabolic alterations such as decreased physical performance, type-2 diabetes, liver fat accumulation, and other chronic diseases, caused by calorie excess. As a strategy to reduce energy intake, non-caloric artificial sweeteners are used to replace sucrose in foods (1, 2). Among them, sucralose (1,6-dichloro-1,6-dideoxy-β-D-fructofuranosy-l-4-chloro-4-deoxy-α-D-galactopyranoside) is a widely used sucrose derivative that is 600 times sweeter than sucrose (3). The U.S. Food and Drug Administration has approved its use in humans and established an acceptable daily intake (ADI) of 5 mg/kg of body weight as safe. However, its metabolic effects are still controversial.

On the one hand, sucralose consumption reportedly associates with negative outcomes in control laboratory animals, such as *D. melanogaster*, in which it exacerbates food intake through activation of the neural fasting response after 5–7 days (4). In lean mice, 8–11 weeks of sucralose treatment alters gut microbiota composition, leading to increased hepatic cholesterol concentration (5) and glucose intolerance (6). In healthy humans, chronic sucralose consumption also alters gut microbiota, which causes glucose intolerance (6). Moreover, in lean albino rats, regular ingestion of sucralose for 1 month causes liver damage (7). In humans, sucralose consumption for 2 weeks decreases insulin sensitivity in healthy subjects (8) and negatively affects glycemic and insulin response after an oral glucose load in obese people (9). However, high doses of sucralose intake do not alter glycemic response in overweight subjects (10). Although these pieces of evidence suggest that sucralose consumption is harmful *per se*, the interaction between sucralose consumption and calorie excess requires further exploration.

The liver is one of the main organs affected by obesity and by xenobiotics. It normally participates in maintaining glucose levels during fasting through glycogen degradation and gluconeogenesis, and also regulates lipid homeostasis through *de novo* lipogenesis and secretion of fatty acids for storage and usage by other organs (5, 11). During obesity and insulin resistance, gluconeogenesis increases, as well as lipogenesis, thereby leading to hyperglycemia and lipid accumulation in the liver (termed non-alcoholic fatty liver disease, NAFLD) and other organs. Of note, hepatic mitochondrial function is critical in these processes, as its dysfunction associates with said alterations as well as NAFLD progression (12, 13). Although NAFLD pathogenesis has been largely studied, it is unknown how it is affected by sucralose consumption. In this work, we aim to study the effect of sucralose consumption on liver metabolism during obesity, using a model of mice fed with a high-fat diet (HFD).

## Methods

### Reagents

All reagents were purchased from Sigma-Aldrich (St. Louis, MO, United States) unless otherwise specified.

### Animals

Male C57BL/6J mice were bred in the animal facility of the Instituto de Nutrición y Tecnología de los Alimentos (INTA), Universidad de Chile, according to the Animal Care and Handling Protocol, approved by the Institutional Animal Care and Use Committee (PT2018-02-FP-RB). The mice were kept under temperature conditions between 21°C and 25°C, 12 h light/dark cycle. 10–12 Animals were randomly distributed to the four treatment groups in the fifth week after birth. The CD (3.82 kcal/g) had a composition of 20% protein, 70% carbohydrate, and 10% fat in total calories (D12450J, Research Diets, New Brunswick, NJ, United States). In contrast, the HFD (5.21 kcal/g) consisted of 20% protein, 20% carbohydrate, and 60% fat in total calories (D12492, Research Diets, New Brunswick, NJ, United States). The intervention spanned 8 weeks, and the mice were given free access to drinking pure water or 0.1 mg/mL sucralose (69293, Sigma-Aldrich) which approximates the FDA-approved ADI in humans.

From the beginning of the study, the food (g), caloric and water (mL) intake, as well as the bodyweight (g) of the mice were determined weekly. After 8 weeks of treatment, the mice were fasted for 6 h, anesthetized with 5% isoflurane, and sacrificed (Zoetis, Parsippany, NJ, United States). A blood sample was obtained from the inferior vena cava, and the hepatic, epididymal adipose tissue, and the soleus and gastrocnemius muscles were extracted and weighed.

### Intraperitoneal glucose and pyruvate tolerance test

Intraperitoneal glucose (GTT) was performed at weeks 4 and 8 of treatment, and pyruvate tolerance test (PTT) at week 8 of intervention. For both procedures, mice were fasted for a minimum of 6 h, with free access to water. They were given an intraperitoneal injection of glucose (2 g/kg body weight) or sodium pyruvate (1.5 g/kg body weight) dissolved in saline. Blood glucose levels were measured from the tail vein at 0, 10, 20, 30, 60, 90, and 120 min using an Accu-Check Performa glucometer (Roche Diagnostic, Mannheim, Germany).

### Treadmill exhaustion test protocol

During the third week of intervention, mice were familiarized with the treadmill (Masterfit, New York, NY, United States) for 10 min for 3 days at 0.3 km/h speed with an incline of 5 degrees. On the last day, 5 min at 0.6 km/h and 5 min at 0.8 km/h were added. After the familiarization period, 5–8 mice performed the test at weeks 3 and 6 of intervention. The trial started at 0.3 km/h for 5 min, and then at increasing speeds by 0.1 km/h every 3 min. The evaluation lasted until voluntary exhaustion, that is, until the animal stopped running.

### Plasma biochemical analysis

Plasma concentrations of GOT/AST (U/L), total cholesterol (mg/dL), c-HDL (mg/dL), and triglycerides (mg/dL) were measured using a Spotchem II Kenshin-2 kit (77188, Arktay, Kyoto, Japan) in an SpotChem Analyzer (Arkray), according to the manufacturer’s instructions. Plasma insulin levels were determined by enzyme-linked immunosorbent assay (ELISA) kit 10-1247-01 (Mercodia, Uppsala, Sweden).

### Intrahepatic triglycerides

Intrahepatic triglycerides were measured using the TG Color GPO/PAP AA kit (Wiener Lab, Rosario, Argentina).

### Hematoxylin-eosin staining

Five to six liver per group were fixed in 10% neutral-buffered formalin overnight at 4°C, dehydrated with ethanol and embedded in paraffin. Paraffin sections were stained with hematoxylin and eosin and visualized under optical microscopy. The analysis of steatosis score was performed according to Kleiner et al. (14), which consists in the evaluation of histological features including semi-quantitative analysis of steatosis, lobular inflammation, hepatocellular ballooning, and fibrosis.

### Western blot

Tissue proteins were extracted using 200 μL of T-PER buffer (Thermo Fisher Scientific, Waltham, MA, United States) in the presence of a protease inhibitor cocktail (Roche, Basel, Switzerland) and phosphatases (Roche) and frozen at −20°C. Protein concentration was determined using a BCA Protein assay kit (Merck, Readington Township, NJ, United States) with a bovine serum albumin standard curve.

Proteins were separated by electrophoresis on 10 or 12% polyacrylamide gels with SDS in a Western Blot chamber Mini-Vertical PAGE/Blotting System (Bio-Rad, Hercules, CA, United States) at a constant potential difference of 80 mV. The Trans-Blot Turbo kit 1704150 (Bio-Rad) was used for protein transfer. Subsequently, the membrane was stained with Ponceau red for 1 min and washed 3 times with TBS-T. Blocking solution (5% semi-skimmed milk in TBS-T) was added for 1 h at room temperature. At the end of the time, the membrane was incubated with primary antibody at 4°C overnight according to the protein of interest: PGC-1 alpha (1:1,000, NBP1-04676, Biologicals Novus, Littleton, CO, United States), mtHSP70 (1:500, #MA3-028, Thermo Fisher Scientific), HSL (1: 1,000, sc-74489, Santa Cruz Biotechnology, Dallas, TX, United States), ATGL (1:1,000, sc-365278, Santa Cruz Biotechnology), OXPHOS (1:1,000, ab110413, Abcam, Cambridge, United Kingdom) and its loading control protein, β-tubulin (1:1,000, #4466, Cell Signaling Technology, Danvers, MA, United States), β-actin (1:1,000, #8457, Cell Signaling Technology), and Vinculin (1:50,000, ab 129002, Abcam). Finally, using Image Studio version 3.1 software, the signal densitometry of the bands was generated. The values obtained for the proteins of interest were normalized by the value of their respective reference protein, thus obtaining relative values.

### Statistical analysis

A two-way analysis of variance (ANOVA) was used for statistical analysis, followed by a Holm-Sidak’s *post-hoc* test for multiple comparisons. Values of *p* < 0.05 were considered significant. Results were expressed as mean ± standard error of the mean (SEM). The statistical analysis was performed using the GraphPad Prism 7 software.

## Results

### Sucralose consumption prevents weight gain in high-fat diet-fed mice

**FIGURE 1:**
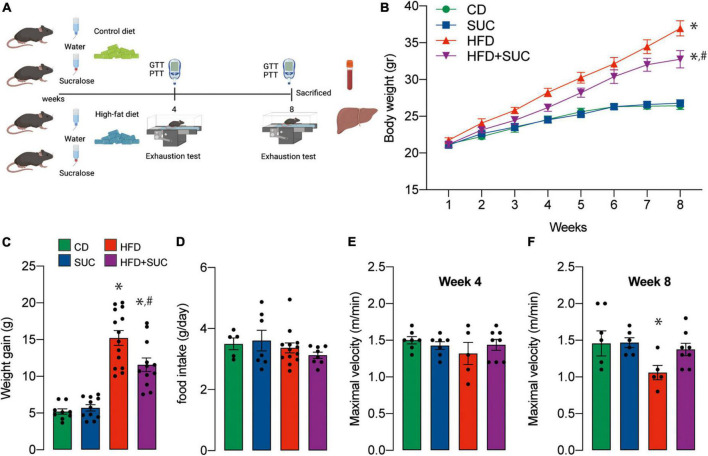
Sucralose reduces body weight gain and improves physical capacity in mice fed with a high-fat diet. **(A)** Study design. **(B)** Body weight. **(C)** Weight gain. **(D)** Food intake. **(E)** Maximal velocity at 4 weeks. **(F)** Maximal velocity at 8 weeks. Two-way analysis of variance (ANOVA) followed by Holm-Sidak’s *post-hoc* test. Values are expressed as mean ± SEM. *p < 0.05 vs. CD; ^#^*p* < 0.05 vs. HFD.

C57BL6/J mice were fed a control diet (CD) or HFD in the presence or absence of sucralose solution (0.1 mg/mL) for 8 weeks (Figure 1A). All animals presented similar body weights at the beginning of experimentation, and as expected, both HFD groups gained more weight than CD; however, starting at week 5, sucralose significantly attenuated body weight gain in HFD mice. This effect was specific for HFD, as no effect of sucralose on the body weight of CD mice was observed (Figures 1B,C). Of note, total food intake after 8 weeks did not differ among intervention groups (Figure 1D). Given the different composition of the two diets, weight gain in the HFD groups is attributable to a higher caloric intake. Interestingly, both HFD groups ingested the same calories, yet the sucralose group gained less weight, which suggests a higher energy expenditure either through physical activity or basal metabolism. To test this, we evaluated physical performance in all groups. The treadmill exhaustion test at week 4 did not show significant differences among groups (Figure 1E). Interestingly, at week 8, the HFD group displayed a significant deterioration in physical state, which was not present in the HFD + sucralose group (Figure 1F). This effect of sucralose on physical state may be due to changes in energy metabolism exerted by the sweetener, or simply due to the prevention of gain weight.

### Sucralose consumption mitigates glucose intolerance and enhanced gluconeogenesis in high-fat diet-fed mice

**FIGURE 2:**
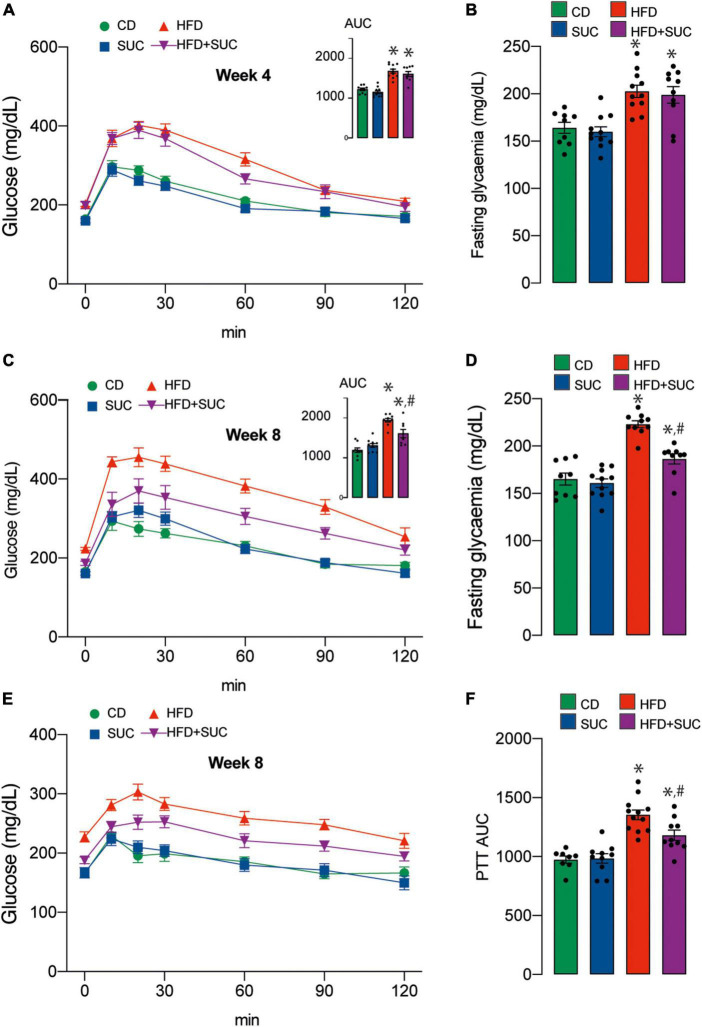
Sucralose improves glucose tolerance in mice fed with a high-fat diet. **(A)** GTT at 4 weeks. **(B)** Basal glucose. **(C)** GTT at 8 weeks. **(D)** Basal glucose. **(E)** PTT. **(F)** PTT AUC. *n* = 8–12 mice per condition. Two-way analysis of variance (ANOVA) followed by Holm-Sidak’s *post-hoc* test. Values are expressed as mean ± SEM. *p < 0.05 vs. CD; ^#^*p* < 0.05 vs. HFD.

At weeks 4 and 8 of intervention, we performed intraperitoneal GTT to determine the effect of sucralose on glucose metabolism. As expected, both HFD groups had significantly higher fasting glycemia and area-under-the-curve (AUC) than CD animals at week 4 (Figures 2A,B). At week 8, however, sucralose reduced fasting glycemia and AUC in the HFD groups. Again, this effect was not observed in the CD groups (Figures 2C,D).

To assess hepatic gluconeogenesis at week 8, we measured intraperitoneal PTT. After pyruvate injection, both HFD groups displayed a higher AUC compared to CD, and sucralose significantly mitigated this effect. No impact of the sweetener was observed on the CD animals (Figures 2E,F). Altogether, these results indicate that sucralose consumption contributes to diminish glucose intolerance and enhanced gluconeogenesis in HFD mice.

**FIGURE 3:**
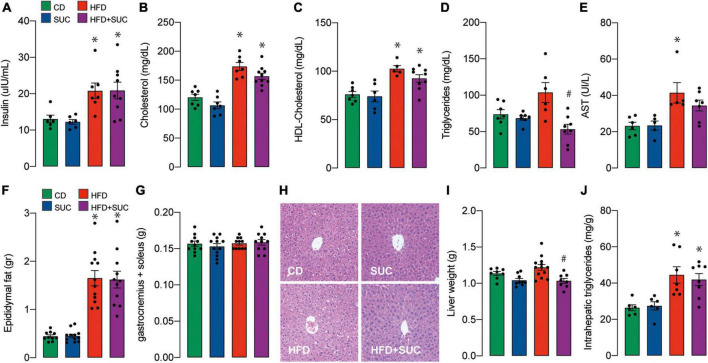
Effect of sucralose on lipid profile, muscle, and adipose tissue. **(A)** Insulin. **(B)** Total cholesterol. **(C)** HDL-cholesterol. **(D)** Triglycerides. **(E)** AST. **(F)** Epididymal fat. **(G)** Gastrocnemius and soleus muscle. **(H)** Hematoxylin-eosin staining. **(I)** Liver weight. **(J)** Intrahepatic triglycerides. *n* = 6–8 mice per condition. Two-way analysis of variance (ANOVA) followed by Holm-Sidak’s *post-hoc* test. Values are expressed as mean ± SEM. *p < 0.05 vs. CD; ^#^*p* < 0.05 vs. HFD.

Plasma concentrations of insulin, total cholesterol, HDL-cholesterol, and were significantly increased in both HFD groups compared with the CD group (Figures 3A–C). On the other hand, triglyceridemia increased in the HFD group compared to CD, which was prevented by sucralose. Again, sucralose did not affect triglyceridemia in CD-fed mice (Figure 3D). As a marker of liver damage, plasma levels of AST were increased in the HFD + water group compared to control animals (Figure 3E). In the case of the HFD + sucralose group, AST levels were not significantly different from HFD-fed nor control mice.

Epididymal fat weight was increased in both HFD groups, while no significant effect on muscle weight (gastrocnemius and soleus) was observed in any group (Figures 3F,G). Consistent with minor liver damage, HFD did not significantly alter liver tissue morphology nor increased liver weight; instead, sucralose induced a relative decrease in liver weight compared to HFD alone (Figures 3H,I). The interpretation of this difference requires further exploration; nonetheless, it is apparently a minor change, because the liver weights of the HFD + sucralose group were not significant compared to the control group. Intrahepatic triglycerides were increased in both HFD groups (Figure 3J). Overall, these results suggest that despite ameliorating gluconeogenesis in a HFD context, sucralose consumption does not impair the onset of steatosis in our animals.

### Sucralose consumption prevents high-fat diet-induced decrease in liver mitochondrial mass

**FIGURE 4:**
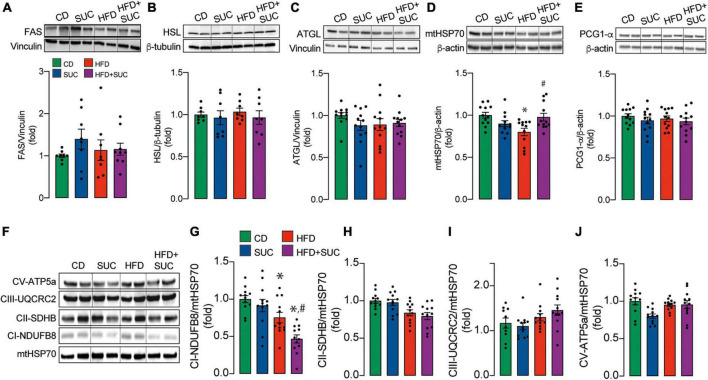
Effect of sucralose on mitochondrial mass and biogenesis. Western blot. Representative image and analysis of the densitometry of the: **(A)** FAS. **(B)** HSL. **(C)** ATGL. **(D)** mHSP70. **(E)** PGC1-α. **(F)** Representative image of mitochondrial complexes. **(G)** Complex I. **(H)** Complex II. **(I)** Complex III. **(J)** Complex V. *n* = 6–8 mice per condition. Two-way analysis of variance (ANOVA) followed by Holm-Sidak’s *post-hoc* test. Values are expressed as mean ± SEM. *p < 0.05 vs. CD; ^#^*p* < 0.05 vs. HFD.

Fatty acid synthase (FAS) is the key enzyme of lipogenesis, while Adipocyte triglyceride lipase (ATGL) and Hormone-sensitive lipase (HSL) are involved in the intracellular degradation of triglycerides. No significant differences were found in the protein levels of these enzymes in liver tissue among any group (Figures 4A–C). In the case of the HFD groups, these results suggest that intrahepatic triglycerides accumulation does not depend on changes in proteins related to the lipogenesis/lipolysis equilibrium, but rather on exogenous lipids intake and/or other regulatory mechanisms.

The marker of mitochondrial mass, mtHSP70, was decreased in the HFD, which was prevented by sucralose (Figure 4D), thereby providing a possible mechanism for sucralose-derived protective effects. The protein levels of the master regulator of mitochondrial biogenesis, PGC1-α, remained unaltered in all groups (Figure 4E), suggesting that the decrease in mitochondrial mass might not be caused by decreased mitochondrial synthesis.

Mitochondrial respiratory complexes in liver tissue, as assessed through western blot, were not altered in any group except for Complex I (Figures 4F–J). Both HFD groups showed a decrease in the protein levels of the Complex I NDUFB8 subunit compared to CD. Of note, HFD + sucralose fed mice showed lower NDUFB8 levels than HFD alone (Figure 4G). Given that mitochondrial respiration participates in fatty acid degradation, these data might explain why the intrahepatic triglyceride levels of HFD-fed mice are similar to HFD-sucralose-fed mice, even though the latter maintain unaltered levels of mitochondrial mass.

Taken together, our results indicate that sucralose reduces HFD-induced body weight and alterations in liver glucose homeostasis while preserving liver mitochondrial mass, but not its oxidative machinery.

## Discussion

Although non-caloric sweeteners were developed to reduce the energy density of foods to avoid weight gain, hyperglycemia, and type 2 diabetes (T2DM) in humans, studies in mice (4-weeks treatment) and overweight/obese humans (12-weeks intervention) have reported that sucralose consumption does not affect body weight, since only a small amount is absorbed in its intact form (15, 16). In our study in mice, sucralose consumption did not affect body weight during the first 4 weeks of treatment; however, in the HFD animals, it attenuated weight gain from the fifth week onward up to week 8. An investigation by Wang et al. (17) showed that 8-week treatment with sucralose did not affect the body weight gain of HFD-fed mice; however, it significantly reduced the body weight of CD-fed mice. The authors point out that the high dose of sucralose ingested (3.3 g/kg/d) could impede nutrient absorption and thus promote weight loss through increased fecal output. In our case, although HFD animals gained more weight than CD mice, no effect of sucralose on food intake was observed, which is consistent with that reported by Wang et al. (17) in terms that the sweetener does not alter appetite. In contrast, it has been shown in *D. melanogaster* that ingestion of a sucralose-mixed diet for 6 days increases appetite and promotes food and caloric intake. The authors note that sucralose causes a sweet/energy imbalance during the post-exposure phase (18). This observation in animals with HFD could be explained by the increased storage of adipose tissue resulting from the high caloric density of the diet, and the high amount of fat ingested (60% of total calories) are a crucial stimulus to generate obesity. Another explanation for the differences in body weight may be due to an effect on energy expenditure in HFD-fed mice. However, that parameter was not measured in our study. Consistently, literature shows that energy expenditure decreases in obese individuals due to decreased physical activity and thermogenesis, which may contribute to energy storage as fat stores. Adipose tissue tends to be metabolically underactive compared to lean mass and therefore has a lower contribution to basal energy expenditure (19).

To determine the effect of sucralose on plasma glucose levels, we performed the glucose tolerance test at weeks 4 and 8 of the intervention. On this topic, primary evidence points to controversial results. Some studies show no effect of sucralose on glycemic control or plasma insulin levels in humans or healthy rats or subjects with diabetes mellitus (10, 20–22). In contrast, in obese subjects who are not regular users of sweeteners, acute exposure to sucralose (48 mg) has been shown to alter glycemic responses to glucose ingestion, producing a greater increase in peak plasma glucose and insulin concentration (9). Unexpectedly, sucralose positively affected glucose homeostasis, decreasing glycemic levels in HFD-fed mice at week 8 of intervention (Figures 2C,D). Qian et al. reported lower blood glucose levels at 30, 60, and 120 min after an intragastric glucose load to Sprague-Dawley rats fed HFD and received 0.78 mM sucralose daily for 4 weeks (23). Even fasting insulin levels were increased in rats exposed to a concentration of 0.54 mM sucralose relative to the control. This phenomenon can be attributed to the activation of sweet taste receptors expressed on β-pancreatic cells, leading to insulin release in response to glucose (24). A study by Temizkan et al. (25) showed that a single dose of sucralose (24 mg) before an oral glucose load in healthy humans increases the release of GLP-1, a hormone with the insulinotropic effect that inhibits glucagon secretion and could be the reason for a lower area under the glycemic curve (25).

HFD-fed mice presented higher levels of intrahepatic TG compared to CD animals, which confirms hepatic steatosis (Figure 3J). Sucralose consumption affected intrahepatic TG levels neither in CD- nor in HFD-fed mice. In contrast, a study in albino rats showed that exposure to sucralose (3,000 mg/kg/day) for 1 month caused hepatocyte degeneration, liver inflammation, and fibrosis (7). We believe that our results differ because of the dosage. In our work, the dose of sucralose was substantially lower (34.7 mg/kg/d for the CD and 26.9 mg/kg/d for the HFD groups) and closer to the FDA-approved ADI in humans, thereby avoiding excessive toxicity. Our differences in sucralose intake between the CD and HFD groups can be attributed to the fact that they are calculated relative to the mean weight of the animals. In C57BL/6 mice, consumption of this sweetener at ∼5 mg/kg/d for 6 months elevated the expression of proinflammatory markers in the liver by altering the intestinal microbiota (26). In our case, we did not evaluate inflammatory markers but liver morphology, which remained unaltered with sucralose consumption. The source of this discrepancy may arise from the duration of the treatment, which in our case was 8 weeks. These observations suggest that sucralose might be relatively innocuous during the first weeks of consumption, but have a long-term toxicity.

mtHSP70 is a mitochondria-resident chaperone protein that belongs to the HSP70 family. It is involved in the correct folding of proteins and their import from the cytosol into the mitochondria (27). PGC1-α, on the other hand, is a transcriptional co-factor that induces mitochondrial biogenesis by activating different transcription factors, which drive mitochondrial DNA transcription and replication. Mitochondrial biogenesis can be defined as the growth and division of pre-existing mitochondria (28). Since mitochondrial biogenesis maintains mitochondrial mass and no changes in the expression of this regulatory factor were observed in this investigation, it suggests that the decrease in mitochondrial mass in HFD mice could be attributed to increased degradation of these organelles and sucralose can prevent this event. However, further investigation is required to address the possible mechanism. Our data agree with other study that reports that HFD specifically reduces the activity of mitochondrial complex I but not that of II or IV, which can lead to a ROS increase and thus contribute to mitochondrial disfunction (29). Like in our work, they also do not observe changes in PGC1-α. On the other hand, in a previous work we reported that in Caco-2 cells sucralose acutely increments mitochondrial function through an increase in mitochondrial Ca^2+^ uptake (30). Because of that, we pose that its effect is mediated through an allosteric mechanism rather than a change in protein expression. In contrast, in the present work, we observe that chronic sucralose consumption preserves mitochondrial mass, but not complex I levels. Given that mtHSP70 is a chaperone, it suggests that it maintains mitochondrial function by preventing an eventual oxidative damage-induced protein misfolding. This potential mechanism also explains the apparent decrease in mitochondrial degradation, which would be due to a preservation of mitochondrial integrity.

Regarding the mechanism of sucralose at the organismal level, our investigation agrees with the results of Qian et al. (23) in showing that it improves glucose tolerance in HFD-fed mice. Also, they report that sucralose increases fasting insulin plasma levels as well as sweet taste receptors in the ileum. Gurmarin, an inhibitor of the murine sweet taste receptors, prevents the protective effect of sucralose. Therefore, they pose that sucralose exerts its actions starting in the intestine through its communication with pancreatic β-cells, stimulating insulin secretion, mediated by hormones like GLP-1. This is similar with the work of Nakagawa et al., which shows that sucralose directly stimulates insulin secretion in the β-pancreatic cell line MIN6 (24).

Among the limitations of this research are that the design of this study is focused on the effect of sucralose on weight gain, not on individuals already obese, therefore, it would be an obesity prevention model. Thus, it does not represent the situation of an overweight individual who adds non-caloric beverages to their diet to prevent further weight gain or as a strategy to lose weight. Also, we assessed the effects of sucralose administration together with the HFD, starting with young mice that were (5 weeks of age) that were still in their maturation period. In other words, we did not only evaluate the effect of sucralose on weight gain but also growth. Finally, our work focused on a time window that showed the early consequences of sucralose consumption, while a more extended study design would allow us to observe its long-term effects.

## Conclusion

Although the health benefits and safety of artificial sweetener consumption remain a topic of debate within the scientific community, our results indicate that continuous dietary supplementation with sucralose for 8 weeks positively affects the body weight and glucose metabolism of mice fed with HFD. In contrast, CD-fed mice are unaffected by sucralose in any of the studied variables. This evidence suggests that sustained sucralose might have a beneficial effect, at least in the early stages of an obesogenic diet.

## Data availability statement

The raw data supporting the conclusions of this article will be made available by the authors, without undue reservation.

## Ethics statement

The animal study was reviewed and approved by the Comité Institucional de Cuidado y Uso de Animales (CICUA), INTA, Universidad de Chile.

## Author contributions

RT and RB-S conceived and designed the study. PP-S, OM, FD-C, FP-d, CD-B and JB performed the experiments. PP-S, OM, RT and RB-S analyzed the data and interpreted the data. PP-S, ML, RT, and RB-S drafted and reviewed the manuscript by all authors. All authors contributed to the article and approved the submitted version.
